# Reaching consensus on factors impacting optimal use of an orthopaedic emergency theatre in a public hospital

**DOI:** 10.4102/hsag.v29i0.2348

**Published:** 2024-03-15

**Authors:** Mamiki Ramokopelwa, Mabitja Moeta

**Affiliations:** 1 Department of Nursing Science, Faculty of Health Science, University of Pretoria, Pretoria, South Africa

**Keywords:** optimal use, orthopaedic emergency theatre, operating theatre, emergency, elective procedures

## Abstract

**Background:**

The operating theatre (OT) complex of hospitals represents areas of considerable expenditure with regard to costs and requires maximum use to ensure optimum cost benefit for both patients and the hospital. Inefficient use of an operating theatre room (OTR) may result in hospital wasteful expenditure and frustrations for patients owing to surgery delays, cancellations and prolonged hospitalisation while waiting.

**Aim:**

The aim of the study was to explore and describe using a consensus method, factors impacting the use of an emergency orthopaedic theatre that can be optimised in a selected public hospital in Gauteng province.

**Setting:**

The study was conducted by a professional nurse and orthopaedic surgeon working in the theatre of a selected public hospital in Gauteng province.

**Method:**

A qualitative, explorative and descriptive design was adopted. Data were collected using a nominal group technique (NGT) among professional nurses and orthopaedic surgeons. Data analysis was done through cross-analysis where participants reached a consensus on the voted ideas from the group.

**Results:**

Consensus was reached and three main themes emerged: (1) inadequate resources; (2) poor organisation and (3) communication.

**Conclusion:**

The optimal use of an emergency orthopaedic theatre is influenced by the availability of resources being human and material, good organisation and clear communication.

**Contribution:**

The study has demonstrated that a variety of factors needs to be considered to optimise the use of an orthopaedic emergency theatre. The management of an OT requires a concerted effort from the nurses and doctors.

## Introduction

The operating theatre (OT) complex of hospitals represents areas of considerable expenditure with regard to cost and requires maximum use to ensure optimum cost benefit for both patients and the hospital to prevent long patient stays, which may result in hospital-acquired infections because of complications associated with surgical delays (Wallace et al. 2021). This is of utmost importance because a major part of a hospital’s budget is spent on OT resources and equipment; hence, they need to be maximally and optimally used (Talati et al. 2014:3) supported by Zhu et al. (2019). An operating theatre room (OTR) functions according to the scheduled patients and the availability of resources, which include material, both human and financial (Ferrari et al. 2011:697). The scheduling of surgical patients depends on their needs, for example, immediate or emergency, urgent, expedited and elective surgical intervention. Patients presenting for emergency surgery represent a category at high risk of complications, with substantial morbidity and mortality, whose management may be extremely challenging and expensive. Accordingly, emergency surgical conditions remain a common reason for hospital admission worldwide (Gray & Morris 2013:3). Operating room (OR) scheduling of patients plays an important role because of the increasing demand for surgical services and the availability of resources.

Management of the OT requires the coordination of human and material resources in such a way that surgery can be performed safely, efficiently and cost effectively (Hassan et al. 2021:23). Shortage of OT personnel that are surgeons, anaesthetist, professional nurses and junior nurses affects the use of an OR, which leads to patients delay, complication and increased length of stay and significant dissatisfaction for patients and relative (De Simone et al. 2023.). According to Moons et al (2019) Operating rooms, in particular, are a costly burden to the hospital as the cost of surgical supplies accounts for 40% – 60% of total hospital supply expenditures. These supply expenses include all items directly linked to the care of patients (e.g., pharmaceuticals, medical consumables, laundry, sterile items, laboratory samples, etc.). Therefore, the OTRs setting appears to be a primary target for material management improvement initiatives and is supported by supply chain processes.

Literature indicates that in South Africa, the most admitted cases in provincial hospitals are related to trauma. About 80% – 90% of trauma and emergency patients present ‘after normal working hours’, which is after 16h00 and may require emergency surgery (Van As, Brey & Numanoglu 2011: 444). After hours, institutions have limited staff, and the number of OR is reduced. If patients are not operated on during normal hours, it becomes a problem to manage the flow after hours because the staff will be limited with few OTRs functioning. Internationally, OT utilisation is measured by the availability of healthcare providers and resources, attitude and good practice (Rashid & Ya’acob 2021). It remains a challenge for OT staff and hospital management to resolve the issue of efficient theatre use and operative delays in relation to operating emergencies, which is exacerbated by inadequate resources (Ifesanya, Ogundele & Ifesanya 2013:423). This trend happens globally, South Africa included.

Worldwide studies have shown that orthopaedic emergency theatres are potentially inefficient in the use of theatre time, therefore, affecting the health outcomes for patients with emergency conditions (Butler et al. 2016:222). A study conducted in Uganda by Kajja et al. (2014) revealed that patients with orthopaedic conditions, who needed emergency surgical procedures were operated on 5 days after admission to the hospital because of inadequate resources, which indicates that there is a delay in operating the patients. Therefore, patients requiring emergency surgery are admitted to the hospital to receive initial resuscitation on arrival, but for some reasons, their procedures are delayed for surgery as expected and are later booked as elective surgeries (Kajja et al. 2014:2). The study conducted in Canada revealed implications of delayed surgery, either in elective cases or in emergency cases, including a higher risk for complications such as nosocomial infections, worsening of the condition, increased length of stay and significant dissatisfaction for patients and relatives (Heng et al. 2012) supported by De Simone et al. 2023. The findings are similar to what is observed in South Africa. Orthopaedic patients tend to be ignored or seen as non-emergency, and this creates problems for patients and the hospitals involved.

### Problem statement

Optimum use of an emergency orthopaedic OTR at a selected public hospital appears to be a challenge. In the selected hospital, both elective and emergency surgical procedures are performed. However, most procedures conducted at the hospital are emergency orthopaedic surgical procedures. Emergency patients are scheduled according to their priority by the surgeon on call from the ward, accident and emergency unit on a 24-h emergency list. The 24-h emergency list is used to schedule emergency orthopaedic patients in a 24-h period from the accident and emergency unit. However, the priority changes as the patients that come from the accident and emergency units are being accommodated as priority owing to the immediate need for surgery. The priority list is marked from one until the last number. If they are 12 on the list number, 12 will fall over to the elective list if not operated within a 24–48-h period. Patients who have not been operated on the 24-h emergency lists are then booked with elective cases but are operated on the emergency theatre. Emergency theatre is occupied with cases that are on the elective list and when urgent cases come the theatre is blocked.

The researcher observed that patients who were not operated immediately are added to the elective list according to the orthopaedic surgical firm that was on call when the patient was admitted. There are different firms (groups of surgeons) in the orthopaedic department, such as spinal, hands and feet and trauma, which normally operates long bone fractures. Patients who were not operated on the 24-h emergency list are added to the elective waiting list; therefore, causing congestion on the elective waiting list and leading to patient frustrations and complications resulting from delayed surgery for both emergency and elective cases (Ifesanya et al. 2013:423). Emergency theatre is occupied with cases that are on the elective list, and when an urgent case comes, the theatre is blocked. After-hours theatre has a limited number of staff and only one theatre is used for emergency orthopaedic procedures.

This creates a backlog and increased length of stay for patients with orthopaedic conditions. The backlog of patients inevitably causes frustration to doctors and nurses in the OR as this is exacerbated by the orthopaedic emergency list being stopped if there is another urgent case from another discipline. Hence, optimising the use of orthopaedic emergency will reduce the backlog and frustration of doctors, nurses and patients. The findings of the study will assist in optimising the use of an emergency OTR.

### Purpose of the study

The purpose of the study was to explore and describe, using a consensus method, the factors impacting the optimal use of an emergency orthopaedic theatre in a selected public hospital.

### Research question

*What are the factors impacting the optimal use of an emergency orthopaedic theatre in a selected public hospital in Gauteng Province?*

## Research methods and design

### Research approach

Qualitative explorative, descriptive design using the nominal group technique (NGT) was used to reach consensus among theatre health professionals on the factors impacting the optimal use of an emergency orthopaedic OT. (Hunter, McCallum & Howes 2019). Nominal group technique, which is a consensus method, was used to collect data from the 12 participants. This technique is one of the most used formal consensuses developmental methods, and Harvey and Holmes (2012) recommend a group of at least 6–12 participants. Nominal group technique was used for reaching consensus while generating many ideas as opposed to other traditional group discussions. A constructivist paradigm was followed to establish the meaning of the topic under study from the participants’ viewpoint (Creswell & Creswell 2018). This approach enabled the researcher to better understand the phenomenon under study from the perspective of the orthopaedic surgeons and professional nurses to obtain first-hand information about their experiences in the use of an orthopaedic emergency theatre.

### Context

The study was conducted in a public hospital in Gauteng province in an urban area. The selected hospital has 21 OTRs, of which the orthopaedics section has 4 allocated theatre rooms. Among the 4 orthopaedic OTRs, 1 is dedicated to emergency orthopaedic procedures while the other 3 are utilised for elective orthopaedic procedures. There are 20 orthopaedic surgeons, including senior and junior surgeons, while there are 19 professional nurses and 1 operational manager for the orthopaedic theatre. The policy of using the emergency theatre room indicates that they must be always vacant, awaiting emergencies. The time schedule for the unit classifies ‘after hours’ as time from 16h00 to 07h00 the next day. After hours, the theatre operates with minimum staff and the emergency cases that were not done during the day are done at night using the emergency OT. Some of the procedures done are Achilles tendon repair, fasciotomies (Compartment Syndrome), ankle, wrist, hand, femur fractures and shoulder dislocations.

### Sampling strategy

Purposive sampling, as a type of non-probability sampling, is a sampling where individuals or groups with special knowledge of the topic are chosen. Burns and Grove (2013:705) also define purposive sampling as a judgemental or selective sampling method that involves conscious selection of certain participants by the researcher to include in the study. The participants in this study were selected because of their knowledge, involvement in the scheduling of orthopaedic procedures and their participation during the performance of orthopaedic surgeries.

Participants had to meet the criteria of working in the orthopaedic OTRs for more than 12 months. The total number of participants, including both orthopaedic surgeons and professional nurses was 12. The purpose and nature of the study were explained and clarity was given to participants.

### Data collection methods

After getting approval to conduct the study, the researcher approached the head of the orthopaedic department for surgeons (DR) and the operational manager for theatre professional nurses (PN) to arrange access to participants and give relevant advice on the best candidates based on inclusion criteria. After access was granted by the orthopaedic head of department and operational manager, the participants were approached. Thereafter, a list of those interested was drafted, with their contact numbers and the date they are available to participate. Consensus was reached on the date and time of data collection. All the participants were on duty on the day of data collection.

### Demographic data of the participants

The study was conducted with *N* = 12 participants, of which *n* = 4 (33%) were DR and *n* = 8 (67%) were PN. (Table 1).

**TABLE 1:** Demographic data of participants.

Participant number	Participant code	Occupation
01	DR1	Orthopaedic surgeon
02	DR2	Orthopaedic surgeon
03	DR3	Orthopaedic surgeon
04	DR4	Orthopaedic surgeon
05	P1	PN
06	P2	PN
07	P3	PN
08	P4	PN
09	P5	PN
10	P6	PN
11	P7	PN
12	P8	PN

PN, professional nurses; DR, doctor.

Data were collected in the orthopaedic lecture room within the selected public hospital, which offered a conducive environment for the discussion away from the OTRs activities. The researcher explained about the consent to be signed. It was also explained to them that they are not forced or obligated to take part in the study, and that the whole process will be audio recorded, which the participants responded by saying yes and some nodded in agreement to participate in the study and signed written consent. Participants who were DR and PN were given white cards to write down their generated ideas from the question, while the research assistant wrote responses on the whiteboard. Research assistant was sought to facilitate the NGT process as the researcher is known to the participants. The NGT lasted about 90 min and was in line with the stages of NGT as described by McMillan, King and Tully (2016:657) and the stages are outlined in Table 2 below. The following stages were followed: Data analysis ran concurrently with data collection using the seven stages of (McMillan et al. 2016): (1) The silent generation of ideas in writing; (2) round-robin recording of ideas; (3) serial discussion; (4) preliminary vote; (5) discussion of preliminary vote; (6) final vote on priorities and (7) listing and agreement on prioritised ideas.

**TABLE 2:** Stages of the nominal group technique.

Stage	Activity
One: The Silent generation of ideas in writing (10 min)	Before the question was asked the researcher introduced the research assistant informed the participants that the data collection process will be facilitated by the research assistant. The researcher gave a brief description of the NGT process to the participants so that they understand how the process will unfold before handing it over to the research assistant Key question was posed and read aloud to participants on the whiteboard. *What are the factors impacting the optimal use of an emergency orthopaedic theatre in a selected public hospital in Gauteng Province?* After the question was posed, research assistant informed participants about the time limit and participants were asked, to each write their views or ideas on a white card, which was provided. Participants were encouraged to write as many ideas as possible about the factors impacting the optimal use of the emergency orthopaedic theatre without discussing them with each other.
Two: Round-robin recording of ideas (15 min)	Participants listed their ideas from the white cards without commenting or discussing until all ideas were exhausted. The research assistant gave each participant a chance to read loud their ideas from the white card and research assistant wrote on the board. The generated ideas were all written on the whiteboard by the research assistant.
Three Serial discussion: (15 min)	The research assistant read each idea that was recorded and gave the participant a chance to discuss a point and clarify the point if necessary. At this stage, some of the points were grouped as meaning the same thing for example, ideas talking shortage of resources either human or material. Ideas that were not applicable or the misunderstanding about the department were clarified immediately for example, stand-alone orthopaedic theatre where participants suggested that orthopaedic theatre should be a standing alone theatre.
Four Preliminary vote: (10 min)	Participants agreed on the ideas that they believe are impacting on the optimal use of an emergency orthopaedic theatre and they were five in number, which was a list they have generated. A list was generated from the discussion and based on the list generated, participants were given clean white cards to write and rank the top five ideas. The participants were asked to select the ideas according to their priority with 1 being most important (5 points) and 5 being least important (1 point).
Five Discussion of preliminary vote: (10 min)	This stage is done simultaneously with the preliminary vote: This phase was incorporated in the preliminary phase as the ideas were discussed during voting. At this stage, the participants have selected their priority ideas and have numbered them from 1 having 5 points and 5 having 1 point.
Six Final vote on priorities: (10 min)	Research assistant wrote the generated list on the board according to their priority and gave the participant a chance to look at the list for any changes. Participants agreed on the final ranking of points.
Seven Listing and agreement on prioritised ideas: (20 min)	The final list was captured on the voice recorder and the board. The researcher also took notes. Consensus was reached among participants on the list and priorities regarding the factors impacting the optimal use of the emergency orthopaedic theatre.

*Source:* McMillan, S.S., King, M. & Tully, M.P., 2016, ‘How to use the nominal group and Delphi techniques’, *International Journal of Pharmaceutics* 38, 655–662. https://doi.org/10.1007/s11096-016-0257-x

NGT, Nominal group technique.

### Data analysis

In using NGT, data analysis is an integral part of the process and therefore was conducted concurrently with data collection. Participants must vote and reach consensus at the end of the session, which was the case in this study. Cross-analysis was used to analyse and formulate the themes from the voted ideas from the group. Cross-analysis is defined as a consensual qualitative method used in developing themes or categories that describe consistencies in the ideas, which were raised by the participants (Van den Berg & Struwig 2017:111). In developing the themes and sub-themes, the voted ideas were grouped to illicit similar meanings and ensure that the voted ideas of the participants were represented

#### Procedure for voting

The number of participants who participated in NGT was 12 whereby they had to rank the top 5 ideas according to their priority. Table 3 depicts the voting of ideas, scoring and ranking.

**TABLE 3:** Results of scoring and ranking of ideas.

Factors	Vote received	Total score	Ranking
Shortage of personnel	2+1+1+2+1+3+1+2+1+1+2+1	54	1
Shortage of material resources	1+3+2+1+3+1+3+1+2+2+5+2	46	2
Poor organisation	4+2+5+3+2+5+2+4+5+3+1+3	33	3
Inadequate patient preparation	5+4+3+4+4+2+4+3+4+5+3+4	29	4
Communication	3+5+4+5+5+4+5+5+3+4+4+5	20	5

Note: For votes 1 is the most important = 5 points and 5 is least = 1 point

### Measures of trustworthiness

The trustworthiness of the study was based on the framework of the Lincoln and Guba (1985) as cited by Polit and Beck (2021). The following trustworthiness criteria were followed: credibility, dependability, confirmability and transferability. Credibility is ensured by presenting the methods and procedures of the NGT thoroughly and clearly, including the voting and consensus reaching. Verbatim quotes from participants are used to support the ideas that were discussed, with subsequent themes and subthemes. Triangulation of data collection tools included audio-recordings and documented transcripts. Also, literature control was conducted on the topic. All the participants were encouraged to freely express their views without fear or intimidation. Data analysis and outcomes were verified by the research supervisors and participants reaching consensus at the end. According to Shenton (2004), it is impossible to demonstrate that the findings and conclusions are applicable to other situations and populations. In this study, transferability was enhanced by using purposive sampling to select information-rich participants who work in OTRs as it is the only single setting that was used and participants will share their experiences that others can relate to outside this public institution. The researcher during data collection ensured that she remained aware of personal biases and own values as part of knowledge and avoided introducing own preferences by keeping field notes and voice recordings to enhance the quality of qualitative data collection. Therefore, the researcher maintained that she approached data in its purest form without the influence of personal values and conflict of interest (Polit & Beck 2021); this enhanced the dependability of the study. Bracketing and use of field notes were used to ensure the researcher reflects about their own biases and to avoid polluting the study findings (Polit & Beck 2021). Therefore, reflexivity contributed to the confirmability of the study. An audit trail was created so that other researchers can be able to verify the credibility of the information in terms of transcriptions, lists of codes, notes and data analysis.

### Ethical consideration

Permission was obtained from the Faculty of Health Sciences Human Research Ethics Committee at the University of Pretoria (471/2018). Additionally, the Provincial Department of Health Gauteng Province approved the study to be conducted (GP_201810_026). Approval was also obtained from the management of the selected public hospital (SBAH_201811_15). The study was conducted in accordance with the guidelines and research principles of beneficence and non-maleficence, respect for human dignity and justice. Participants were informed that their participation was voluntary and could withdraw at any time. Informed consent was obtained, and participants signed an informed consent document to participate in the study. Names of participants were kept anonymous as they were allocated numerical codes even on the transcripts. Data were encrypted with a password and stored on the researchers’ laptop and will be destroyed after 5 years.

## Results

### Themes and subthemes

Three themes and their subtheme that emerged from the findings are: (1) Inadequate resources with subthemes: Shortage of personnel and Shortage of material resources. (2) Poor organisation with subthemes: Readiness of surgical set, Lack of knowledge among theatre personnel, and Inadequate patient preparation. (3) Communication with subtheme: Communication among theatre personnel.

#### Theme 1: Inadequate resources

It emerged from the study that participants strongly expressed their concerns about the lack of resources required for performing smooth operations. Some of the participants were of the view that a public tertiary hospital should be well equipped with resources as it is a training institution, and it caters for complicated conditions. Participants raised a concern about inadequate resources that interfere with their daily activities as it delays the start and completion of surgical procedures, therefore, impacting the optimal use of orthopaedic emergency theatre. Two sub-themes emerged from this theme namely: shortage of personnel and shortage of material resources.

**Sub-theme 1.1: Shortage of personnel:** The participants indicated that a shortage of staff has implications for both day and night shifts. Participants also mentioned that management must ensure that both shifts are well staffed, but moreover, the emphasis was on staffing the night shift. The shortage at night affects the team that is expected to be operating during the night. Participants mentioned that the shortage is not only experienced by one category but all categories including doctors, nurses, porters and central sterilisation and supplies department (CSSD) personnel. This was expressed as follows by one participant:

‘I’ll start from shortage of personnel from both nursing personnel and the doctors. From our side it is obvious that if it’s one sister [*professional nurse*] allocated for the whole list then there will definitively be delays between the cases.’ (08, P4, PN)

According to participants, the shortage of PNs results in one PN being allocated in the theatre to manage the theatre list alone. It causes a delay as the one PN must prepare the theatre, collect sterile sets and look for extra stock; she must do everything herself:

‘Shortage is a problem in our teams because there are other emergencies that need to be attended. Especially at night where there are gynae and obstetrics emergency you can’t do anything about it because other discipline when they got emergencies they are between life and death. So, you actually have to think along those lines because our nursing teams are actually few.’ (03, DR3, Orthopaedic surgeon)

Another participant added that because of staff shortages, staff are rotated to other duties:

‘If there is another emergency, emergency theatre is stopped and the anaesthetist is taken to open another theatre, so that also delays the patient to be operated in the orthopaedic emergency theatre.’ (05, P1, PN)

This indicates that a shortage of staff results in an overload of staff and creates delays. Being few staff on duty, results in patients not being operated on timeously and contributes to the backlog because patients are cancelled. Those on duty end up being exhausted.

**Sub-theme 1.2: Shortage of material resources:** Other participants also indicated that material resources are essential because they also contribute to the smooth running of the emergency theatre. Surgical procedures are dependent on the availability of material resources. If a specific product is not available and it is needed during surgery and there is no alternative, the surgical procedure becomes abandoned. That is a waste of time and unnecessary expenditure as the procedure was not a success. This was expressed as follows:

‘In my experience, a couple of small things that caused us theatre time some of which saving things like cautery, vac dressings and difficulty getting this stock. so, in the end of the day you have a challenge that you need a vac dressing and it’s so scarce to find. It can be frustrating.’ (02, DR2, Orthopaedic surgeon)

The limited resources make it difficult to perform procedures in different theatres. The orthopaedic surgical team must wait for the other theatre to complete the operation first. This was expressed by participant as follows:

‘…like the sister saying, the booking of patient. Sometimes they can book the same procedure in two theatres, and we have only two sets of bone holding forceps after that what’s going to happen… sometimes there is no equipment’s.’ (07, P3, PN)

‘…sometimes when you want e.g. diathermy we don’t get because some of the sisters hide them for themselves so that they don’t struggle.’ (07, P3, PN)

Participants acknowledged their contribution to the shortage of material resources because they hide them so that they could use them when they were on duty to avoid struggling. Those who are left behind to work do not have resources to work with.

Participants voiced that a shortage of resources either human or material affects the smooth running of the orthopaedic emergency theatre. The participants acknowledge that if that can be improved, the operations will be done on time.

#### Theme 2: Poor organisation of the theatre’s daily activities

The poor organisation refers to how the theatre runs every day. Participants expressed in different ways that the organisation of theatre on its own with different levels affects the effective running of the theatres. This poor organisation causes fatigue as most of the time they are looking for items to work with. The three sub-themes emerged from this theme, namely, readiness of surgical sets, lack of knowledge among theatre personnel and inadequate patient preparation.

**Sub-theme 2.1: Lack of the readiness of surgical sets:** Some participants expressed grave concern regarding loan sets from companies. When they are received, they need to be sent to CSSD for decontamination and sterilisation especially if they do not have a proof of decontamination and sterilisation. The loan sets must be sent to CSSD on arrival. If autoclaves are not working, the sets will not be ready:

‘…also mm… regarding loan sets specifically you find that they bring it a day before but most the time it’s late so if they bring it late and they find out that the autoclaves are not working or only 2 autoclaves are working then obviously they cannot sterilise all the sets for the next day on time. So will have to wait in the morning to wait for the sets that we need for specific emergency.’ (08, P4, PN)

This indicates that there is no guarantee that the autoclaves will be functioning, and the staff are used to them not working, and this results in more delays. Participants also raised a concern that loan sets are delivered late and have to undergo decontamination process before sterilisation, which might be delayed by autoclaves that are not working:

‘Surgical sets should be checked before the list starts. They should be sure that all sets are ready and sterile. This should be checked before the case starts and before the whole list for the day and emergency theatre list starts mmm the sets should be ready and sterile.’ (01, DR1, Orthopaedic surgeon)

Participant mentioned that the availability of the sets should be checked before the list starts for electives and also sets should be readily available for emergency theatre. These theatres should be able to work concurrently with their own sets and equipment’s.

Central sterilisation and supplies department plays a major role in the smooth running of the OT and any disorganisation can lead to the OT list being cancelled or delayed. Without the proper functioning of autoclaves, the sterility of equipment is not guaranteed, and operations will not be performed. Therefore, this leads to delayed surgery, which might result in patients developing sepsis and it will further lead to long hospital stay because surgical intervention would have facilitated recovery.

**Sub-theme 2.2: Lack of knowledge among theatre personnel:** Most of the participants expressed a lack of knowledge from other members of the theatre as contributing to delay in the effective utilisation of orthopaedic emergency theatre. The OR is a specialised area that requires the staff to be competent. The surgeons are dependent on the company representatives for guidance on how to use the sets for the successful completion of the procedure. The PNs are not trained on specialised sets; it becomes a challenge because the surgeon also does not know the set:

‘Sometimes you find that you must wait for the medical rep because no one knows the sets. Sometime if the nursing team does go for training for the sets you find that the scrubbing sister is aware of the procedure even though the procedure goes quicker, sometimes you ask for something the sister doesn’t know you end up having to abandon and go look for the set.’ (03, DR3, Orthopaedic surgeon)

‘Qualified staff members must be employed, especially in CSSD, because that’s where we experience problems. They prepare but we also need to prepare again when the sets are here because they are incompletely packed. We run around searching for more things.’ (10, P6, PN)

The participants acknowledge that they do not know the loan sets and they have to wait for a company representative to guide them throughout the operation. They mentioned it would be beneficial if the scrub sister goes for training so that they are able to use the loan sets.

**Sub-theme 2.3: Inadequate patient preparation:** Participants acknowledged that patients are not well prepared for surgical procedures; most of the time the consent form is not completed or signed by the doctor as it is required that the doctor should explain the procedure to the patient. They also mention that the follow-up of blood results is not done prior to surgery, which adds to the delay to start the operation:

‘My first one is consent form not well signed by the relevant people. An orthopaedic patient was brought to theatre and on reception I noted that the doctor did not sign the consent. When I asked the nurse escorting the patient, she said the patient came as an emergency from emergency department.’ (12, P8, PN)

Another participant added:

‘Patients are just wheeled to theatre without proper documentation and nurses claim that it is an emergency. Even if it is an emergency this patient should have been seen by the doctor who decided to perform an operation, therefore informed consent should have been given and be valid for legal reasons. Most of the challenges with informed consent form is failure of the proposing doctor to completely sign the informed consent that is why there are gaps.’ (06, P2, PN)

The participants in this study expressed concern about the inadequate physical preparation of patients by the ward staff. When the theatre sends staff to fetch the patient, the relevant nurses and the porter are delayed by the ward as they will still be doing preoperative care, including giving premedication, collecting blood for diagnostic tests and dressing in theatre attire. One of the participants stated:

‘This one it has been a problem ever since that patients are not prepared properly. When you go into the ward, you will have to look for the patient. When you enquire about the patient for surgery that you came to fetch, nobody knows about the particular patient. Sometimes you will have to wait for the patient to be given premedication.’ (05, P1, PN)

Another participant added:

‘…the patient’s blood results were taken by the anaesthetists and / or doctors and not returned to the patient’s file. When receiving the patient and I check prescribed diagnostic test, I find that blood was collected a day before or earlier that day, but not available on the patient file. When you contact laboratory, they confirm that yes the results have been issued…’ (07, P3, PN)

This indicates that there is no one who follows up on the patients’ blood results and this causes a problem especially if those results have an impact on the decision of what type of anaesthesia to be administered and the surgery itself. This may result in complications that might have been avoided.

#### Theme 3: Communication

Communication in the OT is crucial because the team needs to rely on and trust each other for the safety and smooth running of the orthopaedic emergency room. The theme emanated from the participants mentioning that orthopaedic surgeons do not communicate with the anaesthetist and PNs. They also do not give full reports to each other because most of the time they do not have full information about the patient themselves. One subtheme emanated from this theme, namely: communication among theatre personnel.

**Sub-theme 3.1: Communication among theatre personnel:** The sub-theme emanated from participants mentioning that orthopaedic surgeons do not communicate, or give incomplete information about the patient’s condition to the anaesthetist and nursing personnel. It was expressed by one participant, who said:

‘Another delay is communication between the anaesthetist doctor and orthopaedic doctors you find some of the patients are not discussed with the anaesthetist and then when he or she finally goes to check on the patient then they find out the patient is not fit for theatre whereas we wasted time while we could have done another patient instead of the one who needed to be checked out first and worked out first.’ (08, P4, PN)

Orthopeadic surgeons do not give anaesthetist a full presentation about the patient thus resulting in the anaesthetist going to assess the patient and requesting some test or patient to be stabilised first before coming theatre. Whereas another patient could have been operated:

‘Sometimes when you ask the nurses to fetch the next patient, they delay and when you are ready to push in the next patient, they tell you patient is on the way. At the end you wonder if they did send for the patient or not….’ (04, DR4, Orthopaedic surgeon)

Some participants expressed that when they communicate, they are not heard, and it becomes frustrating as it leads to delays and affects trust:

‘When doctors are on call, they don’t know the patients you find that he doesn’t have the report from the one who’s handing over. So, you will find that patient have ate and some are not in the ward. They don’t know if they are coming to theatre, so it causes delay.’ (12, P8, PN)

Participants expressed that the handing over of patients is not done because the surgeon might not know about the details or updates about the patients on the emergency list. They will only find out when they request that the patient should come to the theatre, that either the patient ate or does not know that he or she is coming to the theatre for operation.

## Discussion

The study findings revealed that inadequate resources interfere with the daily smooth running of an emergency orthopaedic theatre, and it was the first theme identified and a concern for the participants. The selected public hospital in Gauteng is a tertiary and referral hospital that treats patients from Gauteng province and other provinces. It has an influx of patients who typically do not follow proper channels of referrals. This results in overcrowding and depletion of resources. Equipment that is available is being overused to the extent of breakage. Moreover, inadequate staffing and material resources translate into longer waiting times and a perception that healthcare professionals, particularly nurses, are seen as the cause of poor-quality hospital services because they are at the frontlines of the healthcare institutions (Adatara et al. 2018:1773). The availability of either human or material resources is crucial for the delivery of safe adequate patient care. This study revealed that the shortage of personnel in OT is a challenge and affects the optimal use of an orthopaedic emergency theatre. These findings resonate with those of Jarrar et al. (2018:1) that there is a shortage of many health professionals, including nurses, doctors, porters and even CSSD personnel. It was further revealed that the world faces a global shortage of almost 4.3 million doctors, nurses and other health professionals (Aluttis, Bishaw & Frank 2014:2). These health professionals form part of the OT personnel.

Participants mentioned lack of resources as a challenge and that they struggle to get small items that are needed like vac dressings or cautery. These also resonate with findings from the study conducted in one of the public hospitals in South Africa by Moyimane et al. (2017) that the shortage of resources as a discrepancy may be owing to the demands for surgical interventions for trauma patients from catchment hospitals. Rajaguru et al. (2019:2) further echo the findings of a study from the study conducted in Tanzania that most surgical patients consume most of the tertiary hospital budget as they receive patients from referral institutions. Rispel et al. (2018) report that material resources are movable assets that can be wastefully used and can be depleted very quickly. There can be a delay in the procurement of material and equipment owing to challenges in budget allocation and the process of approving the purchases. This is consistent with the study findings as the participants mentioned that because of the delay in procurement and delivery, some of the PNs are locking some of the material resources away to use so that they do not struggle when they are on duty. This results in those remaining struggling because the minimum resources left are being hidden.

The demand for effectiveness and productivity is high in the OT, and such demand can lead to unexpected events, incidents and threats to patient safety. This was the case in this study, where the surgical team is always under pressure to perform their work with maximum efficiency in a minimal amount of time and with limited resources. This was supported by Ingvarsdottir and Halldorsdottir (2017:951) who reported that poor organisation can lead to poor performance of the team, which might lead to increased theatre time and low productivity levels that indicate a problem in an organisation’s structure. Through inefficient resource allocation, poor vertical communication and employee empowerment constraints, employees may not have the proper environment to complete their work assignments in an efficient manner.

The findings of the current study showed that there is a challenge with finding surgical sets before the commencement of the operation. Some participants mentioned that the scrub nurse must run around through other theatres looking for sets and this causes exhaustion and frustration which can be attributed to poor organisation. The study finding resonates with those of Guédon et al. (2016:2729), who confirm that the unavailability of instruments before the procedure result in delays and it is stressful for staff in OT.

Orthopaedic disciplines use loan sets that are ordered per patient use because they are specialised instruments that the hospital does not need to purchase, and the company representative will assist in using the instruments. The company representative is a product specialist who has been trained to use specialised instruments (Grundy et al. 2018:589). The participants’ concern is the delivery time and ensuring their availability before the commencement of the operations. They are delivered unsterile. Outsourcing of orthopaedic loan sets and use of a centralised sterilisation unit is not unique at the hospital where the study was conducted. Guédon et al. (2016: 2729) reported that most orthopaedic sets are obtained from loan companies and should be ordered on time to avoid delays. Central sterilisation and supplies department has its importance in the prevention of Hospital Acquired Infections (HAI) or Nosocomial infections. The department is responsible for cleaning, decontamination and sterilisation of all reusable instruments and supplies (Singh et al. 2019:1264). Singh et al. (2019) further elaborated that any defects in sterilisation can lead to catastrophic consequences and economic burdens which are not limited to increased length of hospital stay, emotional stress, disability, or death of the patient. The challenge arises when the autoclaves are malfunctioning which happens frequently, and it affects the readiness of the sets. It contributes to the delays in an emergency orthopaedic theatre. Outsourcing of sterilisation of instrument in the public hospital where the study was conducted will address the challenges that were mentioned by the participants, and it will present the opportunity of saving costs as supported by the study conducted by Guédon et al. (2016:2729).

The study conducted by Ugur et al. (2016:595) shows particularly that insufficiently trained staff was found to be one of the most common causes of patient safety errors and delays in the OR. This is consistent with the findings of the current study that revealed that the PNs and the orthopaedic surgeons do not know the loan sets. They are both dependent on the company representative (device representative) to come and assist them with instrumentation and waiting for the company representative delays the start of the operation. The participants suggested that orthopaedic surgeon and PN could start the operation while the company representative is on the way to the hospital to assist during surgery with the specialised instruments. The presence of company representatives in OT was confirmed by Grundy et al. (2018:590), asserting that with growing complexities and rapid innovation in medical devices, surgeons increasingly rely on device representatives for guidance during procedures and training on the use of the devices and equipment. Companies upgrade their products frequently, some at the rate of 6 months, which makes company representative the only person who will know how the product or equipment works. (Grundy et al. 2018:590). Guédon et al. (2016:2728) concur that because of the increasing use of technology during surgeries and the complexity of instrumentation, there is an increase in equipment-related incidents. It also increases stress levels especially among orthopaedic surgeons because their surgeries are highly dependent on procedure-specific instruments. Company representatives should be on time for the procedure to avoid delays and come up with instruments that are ready to be used as their expertise is needed on the use of specific instrumentation as alluded by Grundy et al. (2018:590).

The current study also revealed that inexperienced CSSD operators (technicians) who pack and label the instruments without the insight of where those sets are going to be used create more problems as the PNs must look for the instrument in all the theatres which are on different floors. In addition, those who collect them from CSSD do not know which ones are used in their OT floors. According to Singh et al. (2019:1264), one of the functions of CSSD is to distribute or transport sterile supplies to OTs and different wards to ensure that their sterility is not compromised in the process. For the effective functioning of any department, it should be staffed by qualified and knowledgeable people who will best represent the department, attain a good reputation of the department and provide quality patient care as needed. Having a control system in place where instruments are recorded in and out of the CSSD will assist the operators in knowing where to deliver them because they will be delivered at the point of collection. This will lessen the running around done by the scrub nurse looking for instruments before the operation.

Our findings revealed that patients come to OT without signing the consent or incomplete consent forms. It takes time to correct the form because the patient will not be operated on with an incomplete consent form. In healthcare, the term ‘informed consent’ is commonly used. It refers to the permission granted in the knowledge of the possible consequences by the patient in agreement with the surgeon and any health care professional. The doctrine of informed consent originated in 1957, and it is about patient autonomy and self-determination. It was first introduced in South Africa in 1967 but was secured in South African Medical and Health Law Jurisprudence in 1994 (Britz & Le Roux- Kemp 2012:746). This includes explaining the options in the language best understood by the patient, telling them about potential benefits, risks, burdens and any complications including the option to have no surgery (Bajada et al. 2016:1). Obtaining consent from the patient or guardian is not just important but the law requires it. Delay in consent or incomplete consent form result in delayed surgery or patient cancellation. Supported by Denny et al. (2016) Patients who are well informed and prepared for the surgical procedure have better experience and outcome.

Consent plays a vital role in every aspect of medicine and surgery. It facilitates the patient in making informed decisions about their treatment (Jeyaseelan et al. 2010:407). Surgeons can only achieve this by taking their time to explain in detail the importance of the surgery and possible complications that might arise owing to the surgery. Jeyaseelan et al. (2010:407) found that a significant number of consent forms were incorrectly or insufficiently completed. This could not only leave the patient confused about their procedure but also leave the doctor open to litigation, with little in the way of documentation support. Consent forms used in the institution are standardised in such a way that the doctor needs to fill in the blank spaces after explaining to the patient about the procedure. Even with that done, it is still a challenge to completely fill in all the necessary information. Doctors leave empty spaces as though someone else must come to complete it. Other countries such as Wales experience challenges when it comes to informed consent for surgery stating reasons for invalid informed consent such as time constraints, legibility and personal attitude often limits the extent to which the consent form is completed (Bajada et al. 2016:1). However, this does not justify operating patients without valid informed consents, as it is unethical and unsafe for patients, hospital and staff. Health Professions Council of South Africa (HPCSA) booklet 4 (2016:12) supports the current study findings that it is unethical not to have written informed consent because it is important that a written record is available of the patient’s consent and other wishes of the proposed surgery. This helps to ensure later, the understanding between the surgeon, patient and or anyone involved with the care of the patient. Informed consent should be obtained immediately when the decision to operate is taken, and the orthopaedic surgeon can delegate a qualified orthopaedic surgeon or a surgeon with sufficient knowledge about the procedure to be performed to obtain the consent as this is supported by HPCSA booklet 4 (2021:5) as revised.

The study findings revealed that patients are not physically prepared before surgery by ward personnel and that results in delays. When the theatre sends for the patient, the nurses and the porters fetching the patients are delayed by the ward as they will still be doing preoperative care, including giving premedication, collecting blood for diagnostic tests and dressing patients for theatre. Bohmer et al. (2014) also support this finding. Surgical patients’ assessment involves preoperative history taking and physical examination to obtain baseline information from the patient, which is vital prior to surgery. This will help to identify patients with a high risk of complications during and after surgery. Akhtar, MacFarlane and Waseem (2013:317) further supported this. Physical assessment preoperatively is important to identify any risk factors for example, patient identification or to alert theatre staff if a patient has allergies and even pacemaker and specific blood results. Pre-operative preparation is vital to patient safety and is a key nursing role. Careful preparation can minimise anxiety and therefore physical effects and ensure patients arrive in the OR ready for surgery (Liddle 2012:12). The ward manager or shift leader of the ward where the patient is admitted should be notified that the patient will be fetched for theatre to ensure that the patient is ready to be collected. This will ensure that there is accountability for any delays in the wards.

The study findings highlighted that some participants experienced poor or lack of communication among the doctors and nurses within OT and wards, which results in causing unnecessary delays in surgical procedures. Communication assists in the performance of accurate, consistent and easy nursing work, ensuring both the satisfaction of the patient and the protection of the health professional (Kourkouta & Papathanasiou 2014:65). Weller, Boyd and Cumin (2014:149) support the findings of this study and state that failures of OT staff teamwork and communication lead directly to compromised patient care, staff distress and tension. Therefore, this makes a substantial contribution to medical error and is a contributory factor in 61% of patient safety incidents.

The surgical OT is a potentially highly stressed environment where poor communication can lead to fatal medical errors. Most of those surgical errors that contribute to morbidity and mortality can be attributed to communication breakdown. The professionals working in theatre include nurses; doctors; technicians; medical students; nursing students and theatre operators and/or cleaners. Multidisciplinary communication among healthcare professionals plays an essential role in information transfer during operation and has relevance to patient safety (Weldon et al. 2013:1677).

The study also revealed that there is poor communication between theatre personnel for example, orthopaedic surgeons, anaesthetist, nurses, operators and porters. Anaesthetists rely on orthopaedic surgeons for the honest presentation of the patient because in an emergency most of the time the anaesthetist is unable to physically go and assess the patient. If honesty and cooperation are not practised, it may affect the patient outcome in a fatal way. In the study conducted in the United Kingdom, ‘all surgeons acknowledged that there is a lack of interdisciplinary and intra disciplinary communication’ (Nagpal et al. 2012:845). Problems identified with the patients in the preoperative phase are not typically communicated between surgical and anaesthetic team leading to inadequate optimisation of the patient for surgery (Nagpal et al. 2012:845). Randmaa et al. (2014:1) also corroborate the findings of this study. This poor communication results in patient delay and wasteful theatre time as the anaesthetist will be doing test before the administration of anaesthesia, which should have been done by the orthopaedic surgeon. Climate meetings in the OT should be encouraged with the OT staff to voice out their challenges and together come up with strategies to improve workflow. Trust is important in the OT as each person has a responsibility to ensure safe patient care.

## Limitations

The study was conducted in one hospital, and it is not known whether the findings would have been the same in other hospitals, and also that the results were influenced by the context where the doctors and professional nurses were in the same setting and could not openly discuss their challenges with fear of offending one another. Anaesthetist and CSSD personnel were not included, and their views were not heard.

## Relevance to clinical practice

Factors impacting on the optimum use of the OT were uncovered and the results can be used to improve the current situation and improve the future planning of the OTRs. Operating theatre rooms should be supported with provision of resources, which are human and material to ensure uninterrupted services. Adequate material resources should be available for every patient and not be hidden for use by other scrub nurses for their convenience. Emergency orthopaedic theatre should be vacant and ready for an emergency; and if it is used, delays should be avoided because it is an emergency theatre. Informed consent is the right of every patient whether undergoing a surgical procedure or any medical treatment. It should be obtained so that the patient can make an informed decision. Orthopaedic surgeons who are busy in theatre should have the support of the second orthopaedic surgeon who will be able to obtain consent, take blood for investigations and follow up on blood results. Also, ensure that the patient is well prepared for theatre. The support of the second orthopaedic surgeon will also assist in requesting loan sets and sensitising the company representative about the urgency of the operation. Orientation of operators and control system to monitor in and out of sets from CSSD should be emphasised to avoid delaying the commencement of operations because of sets not being found. Communication is the key to have a successful working team and should be encouraged. Operating theatre staff should communicate honestly to each other about the patient to prevent medico-legal hazards and promote patient safety. Regular climate meetings should be encouraged. This will assist OT staff in providing quality care in a reasonable time, thus reducing complications associated with delayed surgery, cancellations and increased length of stay. The patients may have improved health outcomes and be satisfied with the timeous operation. In recommendations made, this study iis aimed at optimising the use of an orthopaedic emergency theatre room. It will also assist the public institution in formulating or adhering to the policy of patient’s admissions to the hospital to avoid overburdening the institution.

## Conclusion

It is evident from the study findings that there are factors impacting the use of an emergency orthopaedic theatre. Participants have identified those challenges and acknowledge that some of them contributed. Shortage of staff results in one PN having to do the list alone, especially at night. Also, frustration about the material resources results in them hiding the little they have so that they do not struggle, which exacerbates the problem. Patient preparation is still a challenge worldwide, and there should be training to emphasise delegation of obtaining informed consent, which is doable and supported by the HPCSA. Company representative should prioritise their patients and arrive on time to avoid delaying the operation. Control systems should be in place to address the challenge of delivering sets in the wrong theatres. Communication is crucial in OT, and OT staff should be able to discuss issues or challenges in the climate meetings, which will improve their morale and workflow.
